# Green Purification of Invertase from Ultrasonicated Sifted Baker’s Yeast by Membrane Filtration: A Comparative Study

**DOI:** 10.3390/molecules30122663

**Published:** 2025-06-19

**Authors:** Serap Durakli Velioglu, Ufuk Bagci, Kadir Gurbuz Guner, Haci Ali Gulec, Hasan Murat Velioglu

**Affiliations:** 1 Department of Food Engineering, Faculty of Agriculture, Tekirdağ Namık Kemal University, Tekirdağ 59030, Türkiye; svelioglu@nku.edu.tr (S.D.V.); kguner@nku.edu.tr (K.G.G.); 2 Department of Food Engineering, Faculty of Engineering, Trakya University, Edirne 22030, Türkiye; ufukbagci@gmail.com (U.B.); ggulec@gmail.com (H.A.G.); 3 Department of Agricultural Biotechnology, Faculty of Agriculture, Tekirdağ Namık Kemal University, Tekirdağ 59030, Türkiye

**Keywords:** invertase, sifted yeast, ultrasonication, membrane filtration, stability

## Abstract

This study aimed to produce invertase with characteristics comparable to commercial formulations using a low-cost raw material, employing environmentally friendly extraction and refinement methods. Sifted yeast, the residual baker’s yeast in industrial production, was used as raw material, and invertase in the yeast cell was extracted by ultrasonication and purified by micro- and ultra-filtration (MF and UF) methods. Subjecting the crude enzyme extract to MF followed by UF resulted in increased activity and specific activity. Through this process, the enzyme activity increased from 153 IU/mL to 691 IU/mL. The purified enzyme was lyophilized and the production of invertase at the laboratory scale was accomplished. The obtained enzyme was found to be stable at pH 5 and within the temperature range of 30–40 °C. It retained its activity for 60 days at −18 °C, whereas a 20% loss in activity was observed at +4 °C over the same period. As a result, it was demonstrated that invertase enzyme can be produced from a low-cost raw material which is considered as waste in the industry. To pass to the commercial production stage, processing of higher amounts of raw material by preventing foaming and heating problems in ultrasonication and scale-up studies testing the permeability properties of different membrane systems at a pilot-scale are necessary.

## 1. Introduction

Invertase, also referred to as sucrase or β-fructofuranosidase (EC 3.2.1.26), catalyzes the hydrolysis of terminal non-reducing β-fructofuranoside residues of several carbohydrates. Also, the transferase activity of invertase (fructosyltransferase, EC 2.4.1.9) can be used in the production of fructo-oligosaccharides (FOS) when high sucrose concentrations are used contrary to acid hydrolysis [1]. Invertase is an eco-friendly alternative to acid hydrolysis of sugar performed at pH 2–3 and temperatures ranging from 70–85 °C causing significant disadvantages, such as the formation of undesired by-products, high energy requirements, and corrosive effects on equipment [2]. Despite its high cost, the use of invertase in the food industry is steadily increasing, particularly in the production of invert sugar syrup [3]. Invert sugar consists of 50% fructose and 50% glucose as a result of the treatment of sucrose (at low concentrations) with either acid or invertase. It is widely used in the production of liquid-filled chocolates and confectionery products, and industrial sweet syrup production due to its enhanced sweetness and non-crystallizing nature during prolonged storage. It is also known as a common product in beekeeping for feeding bees.

Industry’s increasing need for invertase has necessitated not only the search for new sources but also the search for economic isolation and purification methods while commercial production of invertase already exists. Some studies are focusing on increasing the yield, diversifying the enzyme sources, and obtaining invertase with high activity. Invertase production using microbial sources has also been performed [3]. In microbial enzyme production, the focus has primarily been on optimizing the process to cost-effectively propagate enzyme-producing microorganisms because there is a requirement for commercial production of invertase from *Saccharomyces cerevisiae* with low energy [4]. Another important invertase source is plant-based materials such as black mulberries [5], potatoes [6], fruit wastes [7], etc. In these studies, suitable enzyme properties for industrial use were achieved and higher recovery of enzyme activity was reported. However, in the realm of industrial enzyme production, one of the major cost components is obtaining or cultivating the enzyme source [8]. Therefore, the use of plant and animal waste materials to obtain several industrial enzymes has been addressed in the literature [9,10]. The study conducted by Tropea et al. [11] demonstrated that the final medium protein content in *S. cerevisiae* cultivation exceeded 40%, supporting our hypothesis that this microorganism could serve as a valuable enzyme source even when considered as an industrial by-product. However, there is no study in the literature targeting invertase enzyme production using industrial microbial production residuals. Hence, the primary subject of this study is the utilization of sifted yeast, the residue of baker’s yeast (*S. cerevisiae*) in industrial production, which is considered to have partially lost its desired technological properties for bread production.

The production of instant baker’s yeast generates a significant by-product: spent yeast cells and cell debris, often referred to as sifted yeast or yeast waste. Traditionally, this waste material has been viewed as a challenge, necessitating proper disposal methods and management to mitigate its environmental impact. The disposal of yeast waste, if not managed efficiently, can lead to environmental concerns, including the release of organic matter into water bodies and potential ecological disturbances [12]. Sifted yeast, generally used as animal feed, can be utilized as raw material in producing higher-value products due to their valuable nutritional components including enzymes such as invertase.

Invertase is secreted to periplasmic space and cell disruption is an important step in the downstream processing of intracellular invertase, like most of the intracellular products, from yeast [13]. Hydrodynamic cavitation, sonication, and/or high-pressure homogenization can be used to release it. However, these methods differ from each other in terms of efficiency and selectivity. The purification of enzymes from yeast cells can often be a complex and time-consuming process. Traditional purification methods typically involve processes such as precipitation [14], chromatography, or adsorption. While these methods can be successful in enzyme purification, they often require the use of chemical reagents and substantial amounts of water [15,16]. Moreover, these methods may involve complex process steps that can lead to waste production and environmental impacts. Membrane filtration seems to be a modern alternative that can transform the purification process into an environmentally friendly and efficient one [17]. This method involves a physical process that utilizes the selective permeability of a membrane to separate enzymes from other components [18,19]. It offers several advantages, such as minimum chemical use, reduced waste production, and simple application [20].

The present study explores the importance of utilizing sifted baker’s yeast as a sustainable raw material for the production of invertase. The primary goals of this study are to test the applicability of ultrasonication in extraction of intracellular invertase from the sifted baker’s yeast and evaluate the performance (retentate and permeate ratios after filtration steps) of the membrane filtration on the purification of the enzyme extract. The crude enzyme extract isolated using ultrasonication (US) was purified using the three-phase separation (TPS), microfiltration (MF)+ultrafiltration (UF), and three-phase separation (TPS)+ultrafiltration (UF) methods.

## 2. Results and Discussion

### 2.1. General Characteristics of the Baker’s Yeast Sample

The moisture, protein, ash content, pH and water activity (a_w_) values, and the elemental composition of the residual baker’s yeast are given in Table 1. The results indicate that the protein content (53.40%), ash content (4.52%), and pH value (5.44) are in line with the values reported in the literature for single cell protein production results obtained from *S. cerevisiae* [11]. It is observed that the moisture content exceeds the expected range of 6–8% for dry yeast. As previously mentioned, the residual baker’s yeast is obtained from the factory in a clumped form. Residual yeast visually resembles dry yeast and is considered a by-product of dry yeast production. However, during the process of being taken from the dryers, packed into sacks, and stored in the factory environment, it is exposed to conditions conducive to moisture absorption. Under normal circumstances, this product, which is typically used as animal feed, is expected to have a higher moisture content compared to dry yeast, which is considered a reasonable outcome.

It is well known that certain elements can act as activators or inhibitors for enzymes. Therefore, understanding the elemental composition of the residual yeast to be used as raw material is important for the present study. Although the effects of these elements on the final product were not evaluated within the scope of this work, an elemental composition analysis of the raw material was conducted, as it is expected to provide valuable data for future studies. When the elemental composition data obtained in the study were examined, differences from the literature were observed. In the study conducted by Yamada and Sgarbieri [21], it was reported that the potassium, magnesium, and copper values in dry yeast, which was obtained as a commercial product and then subjected to washing, centrifugation, and spray-drying processes in a laboratory, were higher than those found in our study. This difference can be attributed to the fact that the material used in our study, as previously explained, was a factory by-product and was subjected to elemental analysis without undergoing any processing, in accordance with the study’s design.

Preliminary experiments were conducted for the ultrasonication process of disrupting the yeast cells and transferring the cell contents to a buffered medium. The parameter values such as sonication power, duration, and solution volume were optimized using the response surface methodology. The effects of ultrasonication time and power on the extracted protein quantity determined with the experiments are shown in the graph given as Supplementary Material (Figure S1). As a result of this study, the selected parameters were 100% amplitude, 20 kHz frequency, 1000 mL sample volume, and a 60 min ultrasonication duration. During the process, the device was programmed to operate in a 3s on/1 s off mode.

When the results are examined, it can be seen that the protein content, which is approximately 53% in the raw material (Table 1), is 3.34 mg/mL in the supernatant (crude enzyme extract) obtained after ultrasonication of the 10% yeast extract. Considering the dilution ratio, it can be stated that approximately 60% of the protein present in yeast cells is taken out of the cells and, therefore, into the extract.

The calculated values for the protein content and enzyme activity of the crude enzyme extract were 3.34 mg/mL and 153 IU/mL, respectively. The activity value obtained in the current study is significantly higher than the value (2.81 IU/mL) reported for crude invertase enzyme extract obtained from the fermentation medium of baker’s yeast in the study conducted by Sirisatesuwon et al. [22]. On the other hand, Akardere et al. [14] used commercially available bread yeast and expressed the activity value after ultrasonication as 136.5 IU/mL. It should be noted that the raw material used in the present study is a by-product of industrial production and the lack of great care in stages such as collection and storage in the factory environment may result in changes in enzyme activity. Careful control of the temperature increases during the ultrasonication process and paying close attention to phase separation during supernatant recovery could have positively influenced the results.

### 2.2. The Changes in Enzyme Activity and Protein Content After the Purification Processes

The changes in enzyme activity and protein content after the purification processes are given in Table 2. Using the TPS method, the obtained invertase preparation had an activity value of 442 IU/mL. In the case of the invertase preparation obtained through MF, the activity value was 691 IU/mL, while the invertase preparation obtained through the combined TPS+MF methods had an activity value of 1553 IU/mL.

It is evident that the TPS method has been successfully employed for enzyme purification, resulting in a significant increase in specific activity [5]. Our findings corroborate this information, as the processing of the crude enzyme extract using the TPS method led to a remarkable (2653%) increase in the specific activity of invertase (Table 2). Furthermore, the enzyme activity increased from 153 IU/mL to 442 IU/mL. Additionally, subjecting the crude enzyme extract to MF followed by UF resulted in increased activity and specific activity. Through this process, the enzyme activity increased from 153 IU/mL to 691 IU/mL. Subjecting the product obtained from the TPS to UF resulted in a final product with an enzyme activity of 1553 IU/mL. Taking into consideration the potential for commercialization, industrial applicability, and process simplicity, as well as the need to mitigate risks associated with excessive chemical usage in industrial production, it was concluded that the purification of the crude enzyme extract through MF was the most suitable approach for this study. In the TPS method, as previously mentioned, every 10 mL of crude enzyme extract requires 5 mL of butanol and 5 g of ammonium sulfate. As a result of this process, the pipetted liquid containing invertase enzyme is approximately 5 mL. However, this method has several disadvantages, including the difficulty of manual pipetting, the low working volume, excessive chemical usage, and the lengthy process, which collectively make it impractical for commercial production. On the other hand, the method of feeding the crude enzyme extract first through MF and then through UF, while resulting in a lower final product activity value, was preferred in the study to be used for production.

Throughout the study, the following process and yield quantities were observed based on actual values: In the purification of crude enzyme extract to obtain enzyme sample 2 (US+MF+UF), from 1 kg of baker’s yeast, 10 L of crude enzyme extract is obtained, and after MF, 9 L of intermediate product is collected. This quantity is then further processed through a 100 kDa membrane, resulting in 8.1 L of intermediate product containing invertase molecules. To remove sugars and other impurities, this portion is passed through a 50 kDa membrane and collected as a 600 mL liquid enzyme preparation. After approximately 24 h of freeze-drying, it is obtained as 16 g of powdered enzyme. To reach the activity level required by the industry (approximately 5000 IU/mL), the powdered enzyme is diluted with pure water to a concentration of 22 g/L, resulting in a liquid invertase preparation. These values further demonstrate that lyophilization led to an enhancement in enzyme activity. According to this calculation, using 1 kg of baker’s yeast, approximately 720 mL of ready-to-use liquid enzyme can be obtained. When obtaining enzyme sample 3 (US+TPS+UF), approximately 1500 mL of ready-to-use liquid enzyme can be obtained from 1 kg of baker’s yeast. However, during the conversion of this quantity of yeast into liquid enzyme, as detailed above, it would require 5 L of butanol and 5 kg of ammonium sulfate. Considering the commercial potential of the enzyme, this method was not deemed sustainable.

### 2.3. Gel Electrophoresis for Protein Identification

**Figure 1:**
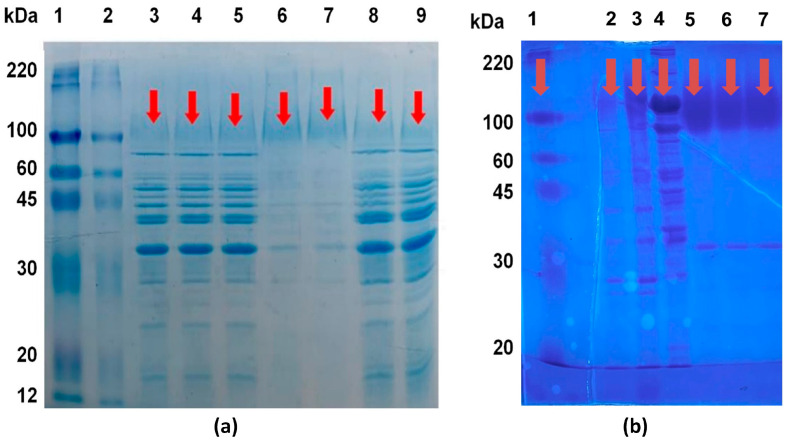
(**a**) Electrophoresis images of enzyme preparations from sifted baker’s yeast. 1–2: Molecular marker, 3–5: enzyme sample 2 (US+MF+UF), 6–7: enzyme sample 3 (US+TPS+UF), 8–9: crude extract (US). (**b**) Electrophoresis images of commercial enzymes and enzyme preparations produced from sifted baker’s yeast. 1: Molecular marker, 2–3: enzyme sample 2 (US+MF+UF), 4: commercial enzyme sample 1 (Liquid enzyme derived from *S. cerevisiae*), 5: commercial enzyme sample 2 (powdered form enzyme sample derived from *S. cerevisiae*), 6: commercial enzyme sample 3 (powdered form enzyme samples derived from *S. cerevisiae*), 7: commercial enzyme sample 4 (powdered form enzyme samples derived from *S. cerevisiae*).

In order to determine the molecular weight of the obtained invertase within the scope of the study and to make comparisons between the preparations obtained from different processes, both with each other and with commercial counterparts, identification was performed using gel electrophoresis. Detection and comparison study was conducted with the presence of a marker, visualizing the MF+UF output preparation (enzyme sample 2), the TPS+UF output preparation (enzyme sample 3), and the crude enzyme extract. The enzyme sample 2 was also visualized in the presence of different commercial invertases (in powder and liquid form, Novozymes) and a marker (Sigma-Aldrich, St. Louis, MO, USA). This visualization is presented in Figure 1a.

When examining the SDS-PAGE (sodium dodecyl sulfate–polyacrylamide gel electrophoresis) results of commercial *S. cerevisiae*-derived invertase samples, it was determined that their approximate molecular weight was 110 kDa. In the context of the study, the SDS-PAGE results of the obtained invertase samples indicated that the invertase in the samples had a molecular size in the range of approximately 100–120 kDa (Figure 1a,b). It is believed that the invertase extract obtained by the TPS method is purer and its activity is higher due to the presence of proteins (invertase) with a molecular weight above 100 kDa. The higher purity of this sample obtained with the TPS method leads to a higher specific activity compared to samples obtained by other methods. As it can be seen from Figure 1a, passing the crude extract, which was not subjected to TPS, through the MF and UF stages did not result in a significant change in the protein profile compared to the crude extract.

### 2.4. Characterization of the Enzyme

**Figure 2:**
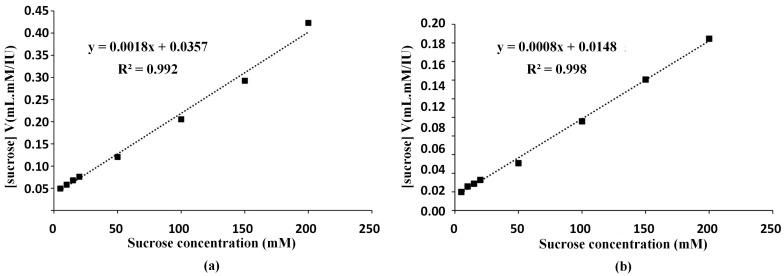
Hanes–Woolf graph of enzyme samples. (**a**) Enzyme sample 2 (US+MF+UF). (**b**) Enzyme sample 3 (US+TPS+UF).

The enzyme sample 2 (US+MF+UF) and sample 3 (US+TPS+UF) were characterized after lyophilization. The Michaelis–Menten kinetic coefficients of V_max_ and K_m_ values were determined from the enzyme activity values according to the Hanes–Woolf approach and the graphs are shown in Figure 2. Using the calibration graph shown in Figure 2a, V_max_ value was determined as 556 IU/mL, and K_m_ value was calculated as 19.83 mM sucrose for enzyme sample 2 (US+MF+UF). For enzyme sample 3 (US+TPS+UF), using the graph shown in Figure 2b, V_max_ value and K_m_ value were determined as 1250 IU/mL and 18.50 mM sucrose, respectively. Andjelkovic et al. [23] investigated the enzyme activity of invertase from *S. cerevisiae* after immobilization. They reported the K_m_ value for free invertase as 26 mM and that of immobilized enzyme as 37 mM. Our result was not only aligned with the literature but also demonstrated the production of an enzyme with a lower K_m_ value, indicating higher affinity of the obtained enzyme towards its substrate.

**Figure 3:**
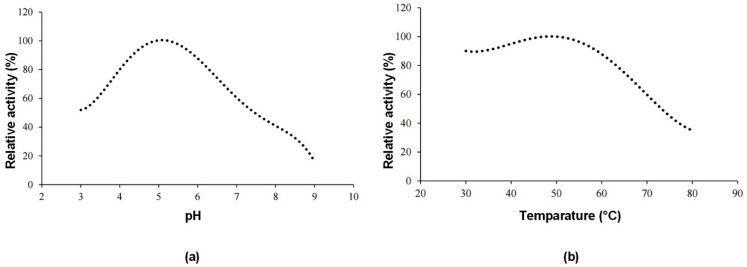
Characterization of invertase enzyme. (**a**) Effect of pH on enzyme activity. (**b**) Effect of temperature on enzyme activity.

Further characterization studies were carried out for enzyme sample 2 (US+MF+UF). Figure 3a shows the effect of pH and Figure 3b shows the effect of temperature on the enzyme activity of the invertase enzyme produced. The optimum pH value was determined as 5, and the optimum temperature was 50 °C.

### 2.5. Stability of the Enzyme

**Figure 4:**
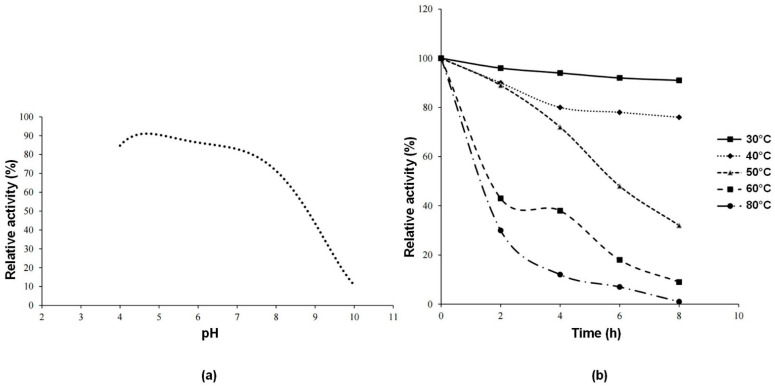
Determination of stability of invertase enzyme. (**a**) pH stability of enzyme. (**b**) Thermal stability of enzyme.

Figure 4a shows the thermal stability of the enzyme after 8 h of incubation. For each pH value, relative activity was calculated by taking the activity of the enzyme at time 0 as a reference. It can be concluded that the enzyme is stable at pH 5. Within the pH range of 4–8, relative activity value is 70% or above that value.

Figure 4b shows the thermal stability of the enzyme during 8 h of incubation in the temperature range of 30–80 °C. The enzyme has high relative activity at 30–40 °C. The results found in this study were slightly higher than the pH 4.5 and lower than the temperature 50 °C reported for invertase from *S. cerevisiae* [24]. The reason for this difference may be attributed to the fact that the invertase enzyme in the referenced study is external, whereas the enzyme in our study is an intracellular enzyme. It is an expected phenomenon for isoform enzymes to exhibit differences in certain characteristic properties [1].

**Figure 5:**
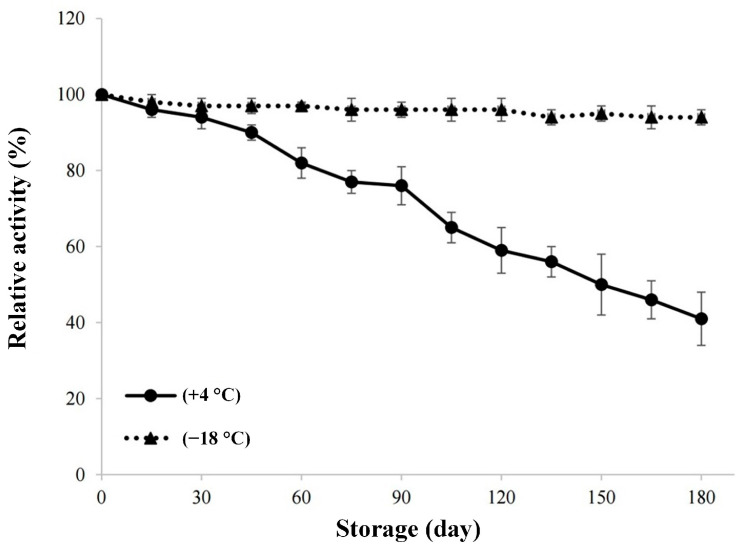
Storage stability of lyophilized enzyme.

For investigating the stability of the enzyme during storage, the graph created using the activity measurements made every 15 days during the 180-day storage period of lyophilized invertase at +4 °C and −18 °C is given in Figure 5. There is no significant change in relative activity value during the storage at −18 °C for 180 days. As it can also be seen from Figure 5, the cold storage of the enzyme would not be enough for long storage periods, because the storage at +4 °C for more than 60 days would result in a relative activity value below 80%. In the study conducted by Andjelkovic et al. [23], the researchers followed the immobilized invertase enzyme activity for 6 months and they found that the enzyme retained 60% of the initial activity. It can be stated that our results are consistent with the aforementioned study.

### 2.6. Microbiological and Elemental Analysis Results

The microbiological analysis results of the lyophilized invertase enzyme preparation produced are given in Table 3, together with the specification data of the commercial equivalent of the product. Microbial criteria for enzyme preparations were described by Food and Agriculture Organization (FAO) as scientific advice of JECFA. According to these limitations, *Salmonella* spp. and *Escherichia coli* should not exist in a 25 g sample, and total coliforms should not be more than 30 per g of sample [25]. Our results are consistent with these criteria.

At this stage of the study, elemental analysis, conducted on the raw material, was repeated both on the crude enzyme extract and the lyophilized enzyme. The data obtained by the ICP-MS method are presented in Table 4. The amount of lead that may be present in enzyme preparations was also specified by JECFA. Accordingly, the maximum allowable lead content in enzyme preparations should not exceed 5 mg/kg [25]. The lead concentration determined in our study (656 µg/kg) is, therefore, considered consistent with the limitations. The elemental analysis of the crude enzyme extract and the lyophilized enzyme sample revealed significant differences, highlighting the impact of the enzyme extraction and purification process on mineral retention and loss. As can be seen from Table 4, the process led to a notable reduction in several elements, including boron (B), magnesium (Mg), aluminum (Al), vanadium (V), manganese (Mn), cobalt (Co), nickel (Ni), zinc (Zn), arsenic (As), selenium (Se), strontium (Sr), molybdenum (Mo), cadmium (Cd), and lead (Pb). Among these, magnesium levels decreased drastically from 644 mg/kg to 27 mg/kg, and boron content dropped from 7583 μg/kg to 1381 μg/kg, suggesting significant losses of essential minerals during the purification steps. Interestingly, potassium (K) and chromium (Cr) concentrations increased slightly. This variation may be attributed to differences in binding affinities, selective retention during purification, or the removal of interfering compounds that could have affected the relative concentrations of these elements.

From a safety perspective, the process appears to have significantly reduced the concentration of toxic metals, such as lead (Pb), cadmium (Cd), and arsenic (As). Notably, lead content dropped from 656 μg/kg to 91 μg/kg, and cadmium levels declined from 668 μg/kg to 19 μg/kg, indicating that the purification and lyophilization steps may contribute to the removal of potentially hazardous contaminants. This reduction is particularly important for enzyme applications in food, pharmaceutical, or biotechnological industries, where toxic element content must be minimized.

Overall, the results suggest that the purification processes altered the enzyme’s elemental composition, leading to substantial reductions in both essential and toxic elements. While the loss of beneficial trace elements may impact enzyme activity and stability, the simultaneous reduction in toxic metals enhances the enzyme’s safety profile.

## 3. Materials and Methods

### 3.1. Materials

The residual baker’s yeast used as the source of invertase in the study was procured from Lesaffre Turquie Mayacılık Üretim ve Tic. A.Ş. (Kırklareli, Türkiye). The clumped portions present in the bulk baker’s yeast were manually broken down using a mortar and divided into 500 g portions, which were then stored in vacuum-sealed bags at refrigerator temperature (+4 °C) until further processing.

Analytical-grade sodium acetate trihydrate, acetic acid, 3,5-dinitrosalicylic acid (DNS), sodium hydroxide, sodium potassium tartrate tetrahydrate, sucrose, Coomassie Brilliant Blue, o-phosphoric acid, and bovine serum albumin (BSA) were obtained from Merck (Darmstadt, Germany), electrophoresis marker was obtained from Sigma (Sigma-Aldrich, St. Louis, MO, USA), commercial liquid and powder invertase preparations were obtained from Novozymes (Copenhagen, Denmark), and ethanol was procured from Labor Teknik (Istanbul, Türkiye) for use in the study.

### 3.2. The Analysis of Baker’s Yeast Sample

The obtained baker’s yeast underwent analyses for moisture content (AOAC 927.05), protein content (AOAC 962.10), ash (AOAC 930.30), and pH (AOAC 981.12). Water activity (a_w_) was determined using a water activity meter (Aqualab 4TE, Decagon, San Francisco, CA, USA). For elemental composition analyses, a modified version of the method recommended by Bilge et al. [26] was employed using an Agilent 7700 ICP-MS (Inductively Coupled Plasma–Mass Spectrometry) (Agilent, Santa Clara, CA, USA). In this procedure, 0.3 g of baker’s yeast samples were subjected to a pre-combustion process in a microwave oven with 10 mL of nitric acid. After filtration, the samples were injected into the ICP-MS instrument (X7, Thermo Electron, Winsford, UK), and the results were recorded. All analyses were performed in three replicates.

### 3.3. Extraction of the Enzyme

In the process of obtaining the crude enzyme extract, firstly a 10% yeast solution was prepared in a buffered medium with a pH of 5.0. The 1 M sodium acetate buffer at pH 5.0 was prepared by dissolving 6.8 g of CH_3_COONa·3H_2_O in 1000 mL of distilled water and adjusting the pH to the desired value by the gradual addition of acetic acid (CH_3_COOH). This solution was stirred on a magnetic stirrer (Wisestir, Seoul, Republic of Korea) for one hour. To disrupt the yeast cells and extract the protein-rich content from within the cells, an HD4200 model ultrasonicator (Bandelin, Berlin, Germany) was utilized. The parameters for the ultrasonication process were determined using the response surface methodology (RSM). The concentration of protein and the activity of the enzyme in the crude extract were the responses against independent parameters such as sonication duration (30–60 min) and amplitude (60–80%). Following optimization, the parameters corresponding to the highest protein concentration and enzyme activity values were selected for further experimentation. The procedure was carried out in an ice bath, and the extract temperature did not exceed 25 °C. After sonication, the extract was centrifuged at 10,000 rpm for 10 min at +4 °C using a centrifuge (Eppendorf 22331, Hamburg, Germany). The protein-rich supernatant, referred to as the crude enzyme extract, was collected. This crude enzyme extract was then analyzed for the determination of protein concentration and invertase enzyme activity.

### 3.4. Protein Analysis

Protein analysis in the samples was carried out using the Bradford method [27]. Bradford reagent was prepared using Coomassie Brilliant Blue G250. A volume of 100 μL of the appropriately diluted sample or 100 μL of sodium acetate buffer solution (0.2 M, pH 5.0 for the blank sample) were added to separate glass test tubes. Subsequently, 3 mL of the Bradford reagent was added to the tubes, and mixed thoroughly. The absorbances of the resulting samples were measured at a wavelength of 595 nm using a spectrophotometer (Mecasys Optizen POP, UV-Vis, Seoul, Republic of Korea). Calibration graphs were prepared using bovine serum albumin (BSA) standard solutions in the 0.1–0.7 mg/mL concentration range, prepared from a 2 mg/mL BSA stock solution. The calibration equation was used to calculate the protein concentrations of the samples using the absorbance values.

### 3.5. Invertase Activity Assay

The determination of enzyme activity in the crude enzyme extract was conducted using the saccharolytic method known as the DNS method. The activity of invertase was determined by means of quantifying the presence of reducing sugars released from sucrose during the enzymatic reaction through the DNS method [28]. One gram of DNS was added to 20 mL of 2 N NaOH solution and dissolved by stirring on a magnetic stirrer. A mass of 30 g of sodium potassium tartrate tetrahydrate were added to a glass beaker with 50 mL of distilled water and dissolved with the help of a magnetic stirrer. The two solutions were combined and mixed on the magnetic stirrer until a homogeneous solution was obtained. The final volume was adjusted to 100 mL with distilled water. The enzyme extract was diluted 1/2000 with the acetate buffer. pH 5 buffer solution, 0.5 M sucrose solution, and the diluted enzyme extract were used for the analysis. The reaction took place in a water bath (JK-WBN-150A, Shanghai, China) at 37 °C for 30 min. During the reaction, the tubes were manually mixed. After incubation, 1000 μL of DNS solution was added to the reaction medium, and it was incubated for an additional 10 min in a water bath filled with boiling water. The mixture was then cooled to room temperature. A volume of 200 μL of the mixture was taken from the tubes and transferred to Eppendorf tubes. A volume of 1000 μL of distilled water was added to each Eppendorf tube, and the mixture was vortexed for approximately 30 s. The presence of reducing sugars was determined by measuring the absorbance at the wavelength of 546 nm using a spectrophotometer. A glucose calibration curve was prepared in the range of 2–12 μmol and used for enzyme activity calculations. The dilution ratio was adjusted if needed. The absorbance values obtained for the samples were substituted into the equation derived from the calibration curve to calculate the glucose concentrations of the samples. In enzyme activity calculations, one unit of invertase activity (IU/mL) was defined as the amount of enzyme that produces 1 µmol of glucose in 1 min at 37 °C, and the enzyme activity was calculated accordingly.

The crude enzyme extract isolated using US was purified using TPS, MF+UF method, and TPS+UF methods. The samples obtained were encoded as enzyme sample 1 (US+TPS), enzyme sample 2 (US+MF+UF), and enzyme sample 3 (US+TPS+UF). All extracts were lyophilized using a laboratory-type lyophilizer.

### 3.6. Triple Phase Separation Method

TPS systems are formed by adding ammonium sulfate salt, which typically causes enzymes of varying molecular weights to precipitate, and an organic solvent such as methanol, ethanol, 1-propanol, 2-propanol, or t-butanol, to the aqueous protein solution [6]. When the necessary conditions are met, the formation of three phases can be observed. In the present study, ammonium sulfate was used as the salt, and t-butanol as the organic solvent to induce protein precipitation. In the resulting three-phase structure, nonpolar compounds were located in the upper phase, ammonium sulfate in the lower phase, and the portion containing protein or the enzyme was positioned in the middle phase.

In the present study, different amounts of ammonium sulfate (20%, 30%, 40%, and 50%) were added to 10 mL of crude enzyme extract in 50 mL Falcon tubes to reach the desired ammonium sulfate saturation. To facilitate the dissolution of ammonium sulfate, vortexing was performed. Subsequently, t-butanol was added to achieve different enzyme/t-butanol ratios (1.0:0.5, 1.0:1.0, 1.0:1.5, 1.0:2.0) [14]. The mixture was allowed to stand for 1 h at room temperature, and phase separation was observed after centrifuging at 4000 rpm for 10 min. The upper phase was carefully pipetted and removed from the medium. The middle and lower phases were collected, and invertase activity was determined using the DNS method, while the protein content was determined using the Bradford method.

Based on the preliminary analysis results, it was decided that the best separation was achieved with 50% ammonium sulfate and an enzyme/t-butanol ratio of 1.0:0.5. For each tube, 10 mL of crude extract was added, and 5 g of ammonium sulfate was added to achieve a 50% saturation ratio, while 5 mL of t-butanol was added for the 1.0:0.5 enzyme/t-butanol ratio.

### 3.7. Membrane Filtration Applications

The crude enzyme extract collected after centrifugation was subjected to membrane filtration in two different ways: (i) the enzyme extract obtained from the triple phase method was fed into UF directly, or (ii) the enzyme extract obtained from coarse filtration followed by centrifugation was first subjected to MF and, then the MF permeate was fed to UF. For both operations, the UF retentate was named as the final enzyme solution.

Laboratory-scale Vivaflow 50 and Vivaflow 200 (Sartorius Lab Instruments GmbH & Co. KG, Goettingen, Germany) membrane modules operating on the cross-flow principle were used in the MF and the UF, respectively. Polyethersulfone (PES) membranes with a pore size of 0.2 µm were used for the MF. For the UF, Hydrosart^®^ membranes with a 100 kDa Molecular Weight Cut-Off (MWCO) (Shanghai, China) were used. The feed was delivered to the membrane module at a flow rate of 10 L/h using a peristaltic pump (Masterflex, Gelsenkirchen, Germany). The feed temperature was maintained at 40 °C using a water bath (DAIHAN Scientific, Gangwon, Republic of Korea). The operating pressure was set to 0.5 bar for the MF using a flow restrictor valve and 2 bar for the UF. The MF process continued until 1500 mL of permeate was obtained from the 2000 mL feed (centrifuged crude enzyme extract). The permeate stream of the MF process and the crude extract solution obtained from the triple phase separation process (900 mL) were subsequently fed into the UF, and this process was continued with a weight reduction factor (WRF) of 5X. Additionally, discontinuous diafiltration (DF) mode was used three times to remove the sugar molecules from the concentrate and to purify the final enzyme solution. In the DF, deionized water was added to the retentate stream when the WRF was reached to 3X. The concentration process was ended when the final WRF was reached to 5X finally. The volume reduction factor was calculated as the ratio of the feed weight ($W_{feed}$) to the retentate weight ($W_{retenatate}$) using Equation (1).

(1) $$WRF=W_{feed}/W_{retenatate}$$

Permeate streams were collected in a container, and the collected permeate amount was measured using an analytical balance (OHAUS Explorer, Nänikon, Switzerland) with a sensitivity of 0.1 g. Samples taken from the feed, retentate, and permeate flows were stored at −25 °C until they were analyzed. After each trial and as needed during breaks in the process, the membrane modules were washed in the following sequence: 15 min with pure water at room temperature, 15 min with 0.5 M NaOH at 40 °C, and 10 min with pure water at 40 °C. Before each trial, the hydraulic permeability of the membrane (with pure water at room temperature) was measured to assess cleaning effectiveness.

### 3.8. Protein Identification by Electrophoresis

In the scope of the study, the invertase preparations were identified by electrophoresis, alongside commercial liquid and powder invertase preparations. The molecular weights of the samples were determined using SDS-PAGE. Electrophoresis gels were prepared using 12% separating and 4% stacking gels. A marker with molecular weights ranging from 8 to 220 kDa was used as a standard.

Lyophilized samples were appropriately diluted to a protein content of 5 mg/mL, and the samples were mixed with “sample buffer” at a 2:1 ratio and subjected to heat treatment at 100 °C for 5 min. After preparation, the samples were loaded onto the gel in a volume of 20 µL, and electrophoresis was carried out at a constant voltage of 100 V. Following electrophoresis, the gel was treated with a solution containing 1% Coomassie brilliant blue in methanol–acetic acid–water (40:10:50 *v*/*v*) for 1 h for staining. After the staining process, the gels were decolorized using distilled water.

### 3.9. Enzyme Characterization

Calculation of Michaelis–Menten constants

To determine the Michaelis–Menten constants (V_max_ and K_m_), a stock sucrose solution with a concentration of 2000 mM was prepared and diluted to appropriate levels. A fixed amount of enzyme was reacted with sucrose solutions of varying concentrations, and the activity values were determined using the aforementioned enzyme activity assay method. Subsequently, the obtained activity/sucrose concentration data were plotted against sucrose concentration, and the slope (1/V_max_) and y-intercept (K_m_/V_max_) were calculated using the Hanes–Woolf linearization method.

Determination of optimum pH

For determining the optimum pH, a fixed amount of enzyme was incubated with 500 mM sucrose solutions prepared in buffer solutions with pH values ranging from 3 to 9 and the activity values were measured. The highest activity value was used as the reference, and the activities at other pH values were expressed as relative activity (%).

Determination of optimum temperature

To determine the optimum temperature, a fixed amount of enzyme was incubated with 500 mM sucrose solutions prepared in buffer solutions with a pH of 5, at temperatures ranging from 30 °C to 80 °C, and the activity values were measured. The highest activity value was used as the reference, and activities at other temperature points were expressed as % relative activity.

Determination of thermal stability

In the thermal stability test, enzyme activity was assessed after incubation at temperatures ranging from 30 °C to 80 °C for 8 h. The pH was kept constant at 5 during the thermal stability test.

### 3.10. Microbiological Analyses

Microbiological analyses including *E. coli* (EN ISO 16649-3) [29], TMAB (EN ISO 4833-1) [30], and mold and yeast (EN ISO 21527-2) [31] enumerations were performed on the final product, the enzyme sample 2. Additionally, coliform bacteria and Salmonella analyses were conducted to allow for a comparison with commercial enzyme specifications, as they were available within the scope of the study.

For TMAB counting, plate count skim milk agar medium was used. The diluted lyophilized enzyme sample was inoculated into petri dishes using the pour plate method and incubated at 37 °C for 48 h. Results were recorded as CFU/g sample. Mold and yeast counts were conducted using PDA medium, adjusted to pH 3.5 with tartaric acid. Samples inoculated in the medium were incubated aerobically at 25 °C, and counts were performed starting from day 3 of the 5-day incubation. Results were recorded as CFU/g sample. For *E. coli* detection, TBX agar medium was used. After preparing appropriate dilutions, samples were inoculated and incubated at 37 °C for 24 h. Blue-green colonies were identified and counted as *E. coli.*

### 3.11. Shelf-Life Study

In the shelf-life study investigating storage stability, the lyophilized enzyme was stored at +4 °C and −18 °C. Enzyme activity was measured every 15 days over a total period of 180 days. Excluding the initial activity, 12 measurements were taken, and a graph of the change in invertase activity over time was generated.

## 4. Conclusions

The present study discusses the efficiency of green purification method of invertase from sifted yeast. The characterization of the final enzyme product based on activity and composition has been also carried out and compared with commercial equivalents. The valorization of spent baker’s yeast for invertase production presents a twofold advantage: It addresses the issue of waste disposal while simultaneously generating a valuable product. By harnessing the biotechnological potential of yeast waste, it can contribute to the principles of a circular and sustainable economy, reducing waste generation and promoting the efficient utilization of resources.

For the commercial production of the enzyme, it is essential to address key challenges such as preventing foaming and overheating during ultrasonication when processing larger quantities of raw material, and conducting scale-up studies to test the permeability of various membrane filtration systems at a pilot scale seems necessary. Further studies should investigate the functional implications of the compositional changes to optimize the purification process for enzyme production.

## Figures and Tables

**Table 1:** Analysis results for residual baker’s yeast.

Analysis	Result ^A^
Moisture (%)	14.35 ± 0.13
Protein (%)	53.40 ± 0.85
Ash (%)	4.52 ± 0.06
pH	5.44 ± 0.04
a_w_	0.64 ± 0.00
K (mg/kg)	13,637.0 ± 171.1
g (mg/kg)	643.9 ± 8.4
Zn (mg/kg)	103.5 ± 1.8
B (μg/kg)	7583.0 ± 64.3
Al (μg/kg)	6167.0 ± 41.1
Mn (μg/kg)	5557.0 ± 43.1
Sr (μg/kg)	4043.1 ± 27.6
Ni (μg/kg)	2988.0 ± 11.3
Cu (μg/kg)	1555.1 ± 7.9
Ba (μg/kg)	609.0 ± 8.1
Li (μg/kg)	503.4 ± 5.8

^A^: Results are given as mean ± standard deviation.

**Table 2:** Changes in enzyme activity and protein content after purification processes.

Samples	Processes ^A^ Applied	Enzyme Activity (IU/mL)	Protein Content (mg/mL)	Specific Activity (IU/mg Protein)	Change in Enzyme Activity (%)	Change in Specific Activity(%)
Crude extract	US	153 ± 22	3.34 ± 0.48	45 ± 0.4	-	-
Enzyme sample 1	US+TPS	442 ± 51	0.37 ± 0.04	1194 ± 11	288 ± 14	2653 ± 8
Enzyme sample 2	US+MF+UF	691 ± 106	4.98 ± 0.62	138.8 ± 4.3	451 ± 18	308 ± 9
Enzyme sample 3	US+TPS+UF	1553 ± 435	6.2 ± 1.4	250.5 ± 13.3	1015 ± 134	556 ± 32

^A^ US: ultrasonication, TPS: three-phase separation, MF: microfiltration, UF: ultrafiltration. The results are given as mean ± standard deviation. -: Not applicable.

**Table 3:** Microbiological analysis results of lyophilized invertase enzyme sample.

	Microbial Load (log cfu/g)	
	Enzyme Sample 3	Technical Specifications of the Commercial Invertase
Total mesophilic aerobic bacteria (TMAB)	2.87 ± 0.41 ^A^	<4.70
*E. coli*	<1	Should not exist
Coliforms	<1	<1.48
*Salmonella*	<1	Should not exist
Total Mold and Yeast	1.88 ± 0.27 ^A^	-

-: information is not available. ^A^: Results are given as mean value ± standard deviation.

**Table 4:** Elemental composition of crude enzyme extract and lyophilized enzyme.

Elements	Crude Enzyme Extract	Lyophilized Enzyme (Enzyme Sample 2)
	Mean (±SD) ^A^	
Lithium (Li, μg/kg)	503 ± 56	493 ± 40
Boron (B, μg/kg)	7583 ± 572	1381 ± 323
Magnesium (Mg, mg/kg)	644 ± 66	27 ± 1
Aluminum (Al, μg/kg)	6167 ± 119	2021 ± 35
Potassium (K, mg/kg)	136 ± 2	151 ± 1
Vanadium (V, μg/kg)	168 ± 9	27 ± 6
Chromium (Cr, μg/kg)	169 ± 32	240 ± 20
Manganese (Mn, μg/kg)	5557 ± 129	786 ± 9
Cobalt (Co, μg/kg)	370 ± 25	18 ± 1
Nickel (Ni, μg/kg)	2988 ± 342	861 ± 67
Copper (Cu, μg/kg)	1555 ± 159	957 ± 16
Zinc (Zn, mg/kg)	104 ± 3	7 ± 0
Arsenic (As, μg/kg)	189 ± 58	83 ± 15
Selenium (Se, μg/kg)	195 ± 76	31 ± 16
Strontium (Sr, μg/kg)	4043 ± 191	242 ± 3
Molybdenum (Mo, μg/kg)	227 ± 22	148 ± 17
Cadmium (Cd, μg/kg)	668 ± 45	19 ± 3
Antimony (Sb, μg/kg)	15 ± 2	12 ± 1
Barium (Ba, μg/kg)	609 ± 19	570 ± 12
Thallium (Tl, μg/kg)	55 ± 6	1 ± 0
Lead (Pb, μg/kg)	656 ± 89	91 ± 3

^A^: Results are given as mean value ± standard deviation.

## Data Availability

The original contributions presented in this study are included in the article. Further inquiries can be directed to the corresponding author.

## Supplementary Materials

The following supporting information can be downloaded at: https://www.mdpi.com/article/10.3390/molecules30122663/s1, Figure S1. The effects of ultrasonication time and power on the extracted protein quantity. Figure S2. Original gel image.

## Abbreviations

The following abbreviations are used in this manuscript:

FOS	fructo-oligosaccharides
US	ultrasonication
TPS	three-phase separation
IU	international unit
MF	microfiltration
UF	ultrafiltration
SDS-PAGE	sodium dodecyl sulfate–polyacrylamide gel electrophoresis
FAO	Food and Agriculture Organization
JECFA	Joint FAO/WHO Expert Committee on Food Additives
DNS	3,5-dinitrosalicylic acid
WRF	weight reduction factor
TMAB	total mesophilic aerobic bacteria
