# Supplementary material for: Green Purification of Invertase from Ultrasonicated Sifted Baker’s Yeast by Membrane Filtration: A Comparative Study
Source: Molecules. 2025 Jun 19;30(12):2663. doi: 10.3390/molecules30122663 (PMC12195727; doi:10.3390/molecules30122663)

Figure S1

The effects of ultrasonication time and power on the extracted protein quantity.

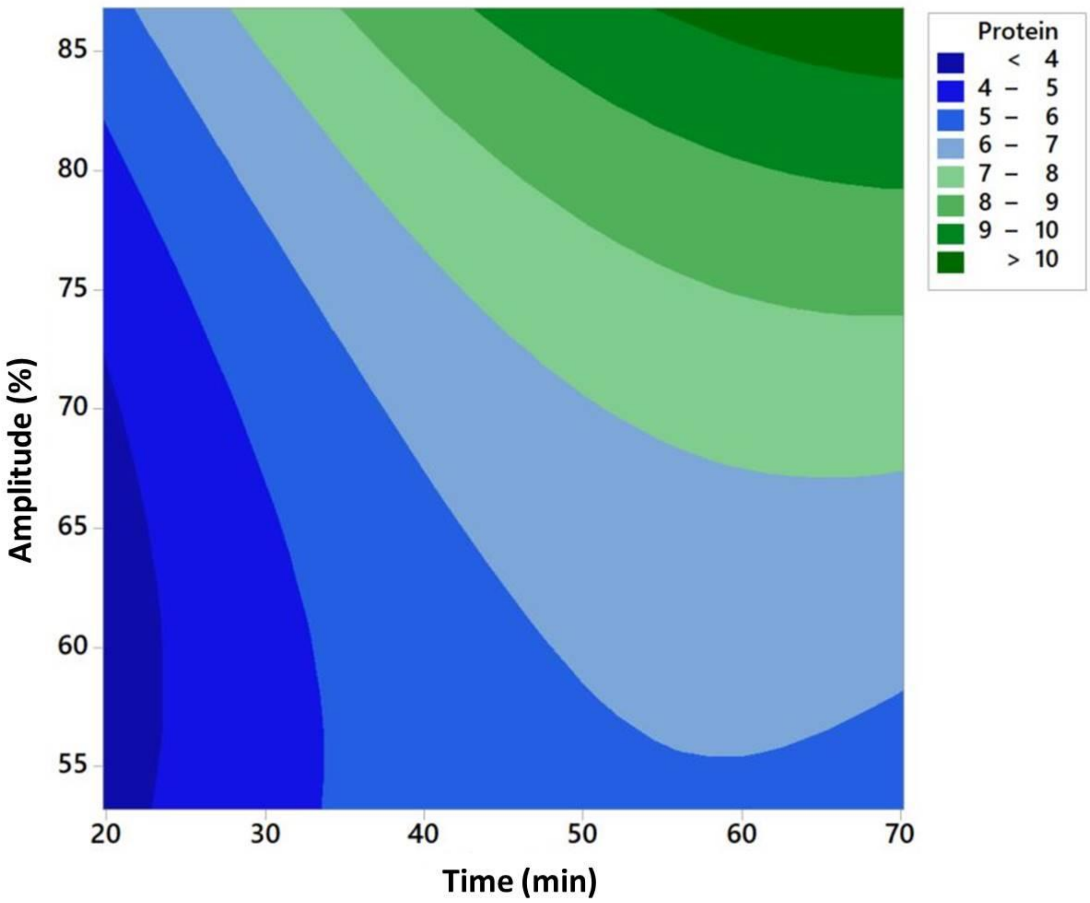

Figure S2

Original gel image

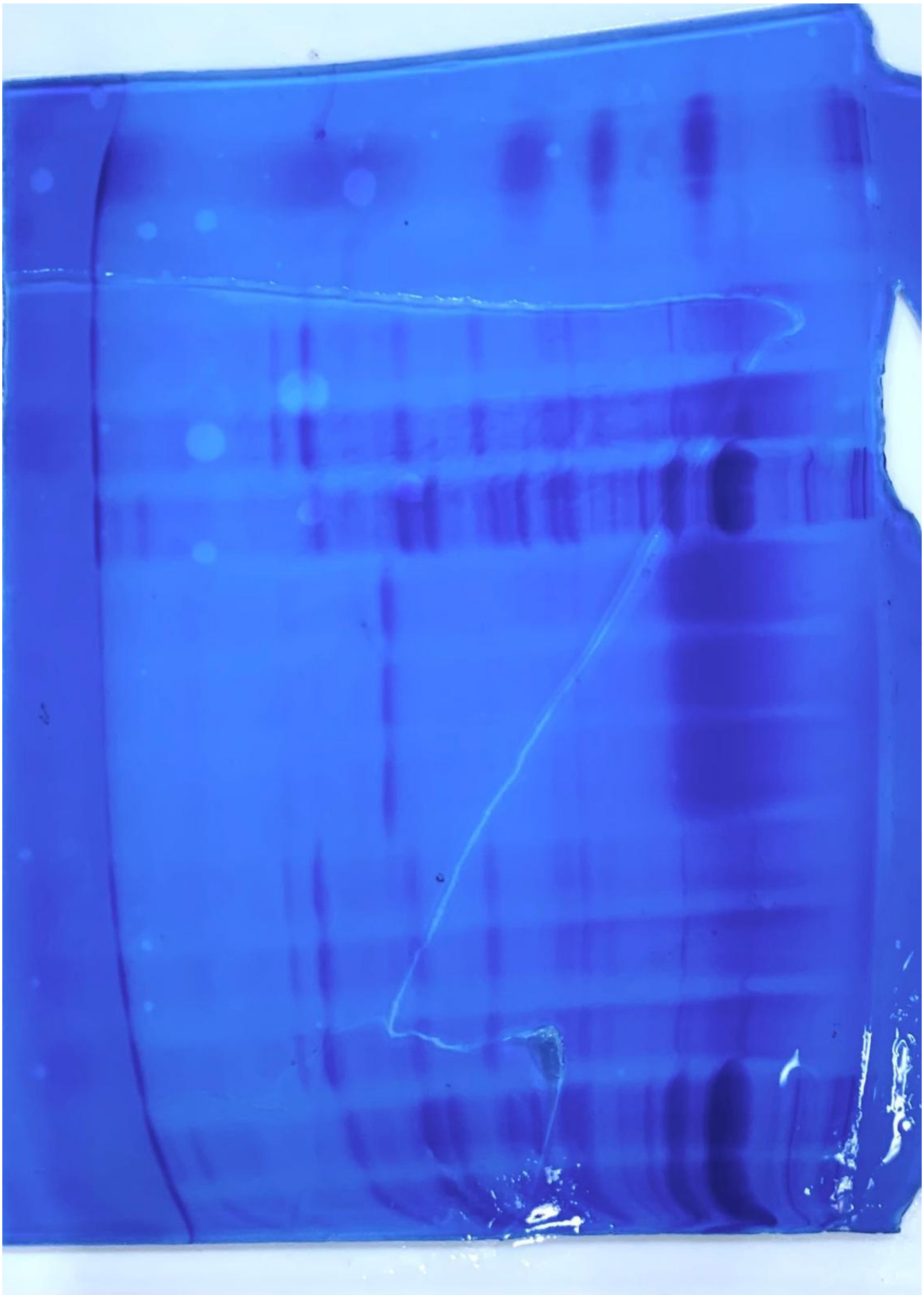

Supplement: Supplementary file 1 [file molecules-30-02663-s001.zip › molecules-3675407-supplementary.pdf]
